# Controlling In Vitro mRNA Polyadenylation by Monitoring Poly(A) Polymerase Consumption of ATP

**DOI:** 10.3390/ijms27072928

**Published:** 2026-03-24

**Authors:** Janja Skok, Pooja Munnilal Tiwari, Tina Vodopivec Seravalli, Sergeja Lebar, Ana Ferjančič Budihna, Anže Martinčič Celjar, Polona Megušar, Matija Povh, Nina Mencin, Swapnil Bawage, Shree R. Singh, Artem Badasyan, Rok Sekirnik

**Affiliations:** 1 Sartorius BIA Separations d.o.o., Mirce 21, 5270 Ajdovščina, Slovenia; 2 Arnav Biotech, 380 Michel St. NW, Atlanta, GA 30313, USA; 3 Materials Research Lab & School of Science, University of Nova Gorica, Vipavska 13, 5000 Nova Gorica, Slovenia

**Keywords:** mRNA, HPLC, poly(A) polymerase (PAP), polyadenylation, poly(A) tail, in-process analytics

## Abstract

The poly(A) tail of mRNA plays a vital role in mRNA transcript stability, translational efficiency, and immunogenicity. Co-transcriptionally polyadenylated in vitro transcribed (IVT) mRNAs typically contain poly(A) tails of 50–120 nucleotide tail length due to limitations in production of template pDNA with longer poly(A) sequences. In contrast, post-transcriptional enzymatic polyadenylation of mRNA with poly(A) polymerase (PAP) presents a modular alternative to increase the tail length. However, the lack of real-time control strategies for PAP-mediated tailing has limited its broader applicability in mRNA production. Here, we develop a methodology for controlling poly(A) tail length in post-transcriptional polyadenylation of mRNA that uses adenosine triphosphate (ATP) consumption measured at-line to predict the poly(A) tail length. We establish a novel analytical method based on monolith reverse-phase chromatography to validate the poly(A) predictions. We were able to produce longer poly(A) tails and accurately determine their length in 300–700 nt range. The resulting longer poly(A) tailed reporter mRNAs outperformed the encoded and shorter poly(A) tailed mRNAs in cell-based assays. This work presents a new strategy for controlled post-transcriptional polyadenylation using ATP consumption as a process control metric, an approach which may in future be expanded to other NTP-dependent enzymatic conversions.

## 1. Introduction

Synthetic mRNA has emerged as a promising programmable therapeutic modality, enabling transient and tunable protein expression that can be harnessed for development of prophylactic vaccines, gene editing, protein replacement therapies and immunotherapies [1,2,3,4,5,6,7]. From the success of mRNA-based prophylactic vaccines, the field has progressed into the mRNA 2.0 era, which prioritizes the enhancement of expression control, safety, and manufacturability. Even though mRNA design elements like UTR optimization and tissue-specific expression are prioritized [8,9,10,11], adjusting structural elements such as the poly(A) tail remain essential. Both the 5′ cap and the poly(A) tail regulate mRNA translation efficacy [12]. While efficient capping mechanisms have been developed to improve mRNA-based therapeutics and vaccines [12,13,14,15], installing and controlling poly(A) tails remains a challenge.

Recent studies have examined how variations in tail length and the choice of alternative polyadenylation sites influence translational regulation, affecting both mRNA stability and translation efficiency [16]. Longer poly(A) tails (e.g., 250 bases) increase mRNA stability and translation by promoting the action of poly(A)-binding proteins (PABPs), which facilitate efficient protein expression and ribosome recruitment [12,16,17,18]. However, cells can dynamically adjust poly(A) tail lengths to modulate translation rates, enabling targeted control over mRNA stability and protein production [19,20]. Most mRNAs degrade after their poly(A) tails are reduced to below 25 nucleotides [21]. Advances in sequencing technologies continue to uncover new complexities in poly(A) tail regulation, reinforcing its significance for enhancing mRNA medicines. Initially thought to promote translation and prevent mRNA degradation, recent research shows that poly(A)-binding protein can enhance deadenylation, and stable, highly translated mRNAs often have shorter poly(A) tails than expected [22]. Improvements in mRNA stability and translation have been observed not only through the optimization of tail lengths but also through other mRNA tail modifications, such as incorporation of multiple synthetic, branched poly(A) tails [23]. Thus, the poly(A) tail is no longer a static design element but a dynamic and changeable feature that can be optimized to balance translation kinetics, immunogenicity, and mRNA stability.

In therapeutic mRNA manufacturing, poly(A) tails are typically introduced either by transcription from a plasmid-encoded tail signal or post-transcriptionally, using enzymatic polyadenylation. Recently, tail modifications have also been reported, including the use of tail mimetics to enhance mRNA expression [24]. Template-based methods often suffer from plasmid instability or recombination, especially with long homo-polymeric A/T sequences. To overcome this, a segmented poly(A) tail with two 40–60 poly(A) stretches separated by a spacer can be used, but that may complicate manufacturability [25].

Enzymatic polyadenylation is an attractive alternative due to its ability to produce long poly(A) tails, but there is a lack of robust tools for real-time control or prediction of tail length. Conventional approaches to measure poly(A) tail length primarily include sequencing-based assays [18,26,27,28], ligation-mediated poly(A) test [29], RNase-H digestion [27] or RNase T1 cleavage of poly(A) tail followed by liquid chromatography (LC) [30,31]. These analytical approaches are accurate and sensitive, but also highly complex, endpoint, and often require the knowledge of RNA sequence for probe design and therefore do not apply generically or to an unknown mRNA sequence.

Based on our previous work on monitoring nucleoside triphosphate (NTP) consumption in the in vitro transcription (IVT) reaction to control IVT yields [32,33,34,35], we have envisioned that at-line monitoring of ATP consumption could similarly be used to control the polyadenylation reaction. We therefore developed an analytical approach to control the post-transcriptional enzymatic polyadenylation of mRNA with poly(A) polymerase (PAP) based on rapid ATP monitoring, coupled with modelling to predict the poly(A) tail length as a function of the ATP and mRNA concentrations. To verify predictions of poly(A) tail lengths, we developed an ion-pair reverse-phase chromatography (IP-RPC) analytical method able to separate RNA chains by size. We demonstrate the use of the new IP-RPC analytical method to analyze poly(A) tail lengths of the commonly used green fluorescent protein (GFP) and luciferase (Luc) reporter mRNA constructs. We further assess the robustness of the approach with a new VHH (variable heavy chain domain of a heavy chain antibody) based multi-tag mRNA reporter construct Decorator^TM^ (mDeco, Figure S1), all polyadenylated post-transcriptionally.

## 2. Results and Discussion

### 2.1. Poly(A) Tail Length Calculation

We built a simplified model of mRNA polyadenylation based on the following assumptions:

1. The number of mRNA molecules in solution is constant;
2. All mRNA chains grow in length unidirectionally from 5′–3′ at the same (average) rate, without accumulation of covalent intermediates [36,37];
3. The length of mRNA changes only due to the incorporation of ATP into the chain.

Condition 1 is satisfied, since there is no degradation or synthesis of mRNA during the PAP reaction.

Condition 2 is an approximation. We consider an average growth rate due to: (i) the adsorption/desorption equilibrium with respect to the enzyme; (ii) very low PAP/mRNA ratio (1:15 to 1:150); (iii) PAP-mRNA adsorption/desorption is a fast diffusion-limited process, while nucleotide addition is a catalytic chemical reaction which is at least an order of magnitude slower.

Condition 3 is a consequence of the mass-balance principle and the reaction pathway.

Polyadenylation is irreversible, such that each ATP serves as a building block of the growing polyadenylic chain attached to a mRNA molecule. In order to determine the reaction rate constant k, a system of differential equations describing the kinetics of reaction can be constructed. Since in each step one molecule of ATP is consumed to add one unit to a growing poly(A) tail attached to mRNA chain at a time, we can consider the reaction as a first order. In order to account for mass balance, we write down the equations in terms of mass concentration $\left[\rho \right]=\left[M*C\right]=\frac{g}{mol}\frac{mol}{L}=\frac{g}{L}$, where M is the molecular weight and C is the molar concentration. Thus, the time-dependent mass concentrations are:

(1) $$\rho_{ATP}\left(t\right)=M_{ATP}*C_{ATP}\left(t\right)$$

(2) $$\rho_{mRNA}\left(t\right)=M_{mRNA}\left(t\right)*C_{mRNA}=M_{ATP}*N_{tail}\left(t\right)*C_{mRNA}.$$

Here we used Condition 1 (the constant number of mRNA molecules in solution) to set the molar concentration $C_{mRNA}=const$ independent of time. The tail length is measured in repeat units and is equal to the difference between the actual chain length of mRNA and the chain length before the start of the reaction: $N_{tail}\left(t\right)=N_{mRNA}\left(t\right)-N_{mRNA}\left(0\right)$. Mass concentration $\rho_{mRNA}\left(t\right)$ is increasing because of poly(A) tail growth. Dividing mass concentrations by the molecular weight of a monomer $M_{ATP}$, leads to

(3) $$x_{1}\left(t\right)=\rho_{ATP}\left(t\right)/M_{ATP}=C_{ATP}\left(t\right)$$

(4) $$x_{2}\left(t\right)=\rho_{mRNA}\left(t\right)/M_{ATP}=N_{tail}\left(t\right)*C_{mRNA}$$

Which were used as the variables of the study. In Equations (3) and (4) and later, index 1 corresponds to ATP-related quantities, and index 2 to mRNA. Chemical kinetics equations describing the change of material quantities with the account of the Conditions 1, 2 and 3 can be written as

(5) $$\left\{\begin{array}{c} \frac{{dx}_{1}\left(t\right)}{dt}=-{kx}_{1}\left(t\right);x_{1}\left(0\right)=x_{0}\\ \frac{{dx}_{2}\left(t\right)}{dt}={kx}_{1}\left(t\right);x_{2}\left(0\right)=0, \end{array}\right.$$

where k is the reaction rate constant and $x_{0}=C_{ATP}\left(0\right)$ is the molar concentration of ATP monomers before the start of the reaction. The solution of the Initial Value Problem (IVP) in Equation (5) is

(6) $$\left\{\begin{array}{c} x_{1}\left(t\right)=x_{0}exp\left(-kt\right)\\ x_{2}\left(t\right)=x_{0}\left[1-exp\left(-kt\right)\right]. \end{array}\right.$$

Taking into account Equation (6), from Equation (4) we can express the mRNA tail length as

(7) $$N_{tail}\left(t\right)=x_{2}\left(t\right)/C_{mRNA}=\left(x_{0}-x_{1}\left(t\right)\right)/C_{mRNA}$$

and compare with values measured after the reaction. From Equation (7) we determined the maximal tail length at infinite time (so-called ‘steady state approximation’). In practice, the steady state is achieved on timescales much longer than the reaction half-life [38], $t\gg t_{1/2}=ln\left(2\right)/k$, when ATP is consumed and the reaction stops, leading to the maximal value of tail length

(7a) $$N_{tail}\approx C_{ATP}\left(0\right)/C_{mRNA},$$

Which is the number of ATPs per each mRNA chain. Although simple, Equation (7a) is critical, since it allows to tune/program/control the length of poly(A) tail in a PAP reaction.

According to the IUPAC Gold Book [39] definition, the reaction rate v for a chemical reaction occurring in a closed system at constant volume, without a build-up of reaction intermediates, is defined as $v=\frac{-{dx}_{1}\left(t\right)}{dt}=\frac{{dx}_{2}\left(t\right)}{dt},$ which using Equation (6), becomes

(8) $$v\left(t\right)={kx}_{0}exp\left(-kt\right).$$

From Equation (8), it follows that the rate constant *k* is related to the reaction rate at zero time and is inversely proportional to the initial ATP concentration:

(9) $$k=v\left(0\right)/x_{0.}$$

The objective of our study was to investigate the route of the PAP reaction and to propose the controlled polyadenylation of mRNA on the basis of a rapid at-line LC-based monitoring of ATP concentration.

**Figure 1:**
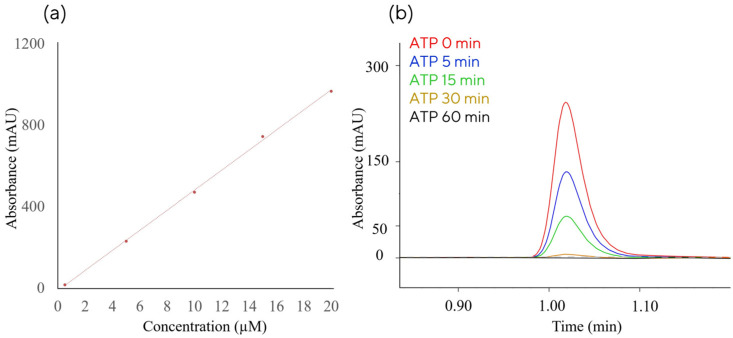
Rapid chromatographic quantification of ATP concentration facilitates at-line measurement of ATP consumption in PAP reaction. (**a**) Calibration curve for ATP concentration; (**b**) analytical chromatographic analysis of ATP from PAP reaction (sampled at 0–60 min) analysed with CIMac PrimaS analytical monolith.

We first verified the ATP concentration response using LC analytics previously used for IVT monitoring [32,33,34,35] and found it to be linear for the concentration range of at least 1–20 µM (Figure 1a). Retention time of ATP peak with the chromatographic method is ~1 min (Figure 1b), which enables very rapid monitoring of the ATP concentration, i. e., the ability to measure the ATP concentration during PAP reaction within minutes of sampling.

**Figure 2:**
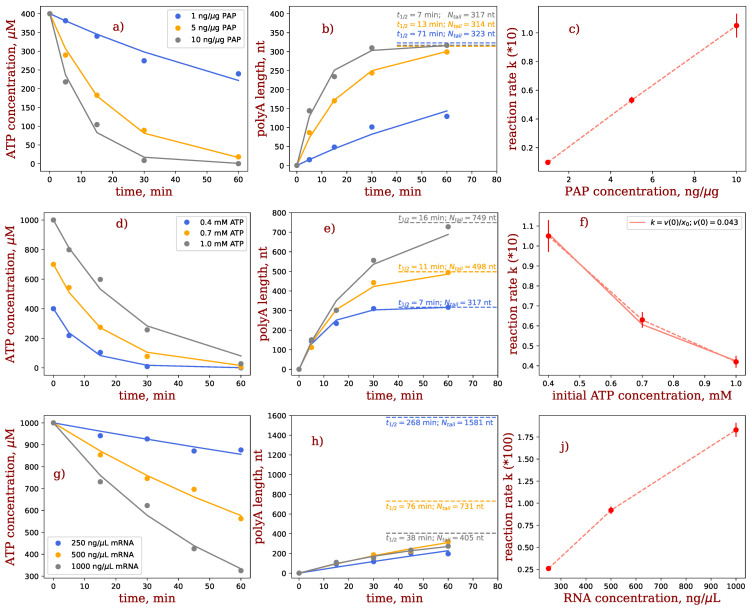
Studying PAP reaction as a function of the PAP (**a**–**c**), ATP (**d**–**f**), and mRNA (**g**–**i**) concentrations. Dots in (**a**,**d**,**g**) are the results of measurements, while lines are fitted *C_ATP_*(*t*) = *C*_0_ *exp*(−*kt*) (see Equation (6)). Dots and lines in (**b**,**e**,**h**) are recalculated as *N_tail_*(*t*) = (*C*_0_ − *C_ATP_*(*t*))/*C_mRNA_* (see Equation (7)). Dashed lines show the tail length at infinite time (Equation (7a)), and *t*_1/2_ = *ln*(2)*/k* is the half-life. In (**a**–**c**) PAP concentration is 1–10 ng/μg PAP at constant ATP (0.4 mM) and mRNA (0.5 mg/mL) concentrations; in (**d**–**f**), the initial ATP concentrations are 0.4–1 mM at constant PAP (10 ng/μg) and mRNA (0.5 mg/mL) concentrations; in (**g**–**i**), the mRNA concentrations are 250–1000 ng/μL at constant PAP enzyme (1.5 ng/μg) and ATP (1 mM) concentrations; dashed lines in (**b**,**e**,**h**) indicate the maximal possible lengths with corresponding half-lives, error bars show the error of fit for the reaction rate *k*.

Next, we experimentally investigated the effect of the polyadenylation reaction conditions on the ATP consumption and poly(A) tail length by varying the concentrations of PAP enzyme (Figure 2a–c), ATP (Figure 2d–f) and mRNA (Figure 2g–i). Measured time dependencies of ATP concentrations (Figure 2a,d,g) were fitted to theoretical results (Equation (6)), obtained from solving the differential equations which describe the reaction kinetics. mRNA concentrations (Figure 2b,e,h) were recalculated using $x_{2}\left(t\right)$ expression (Equation (6)), both point-by-point from the ATP measurements (dots), and using the fitted values (solid lines). Reaction rate constants (Figure 2c,f,i), derived from the fitting procedure, revealed the dependence of polyadenylation on reaction conditions.

### 2.2. Impact of PAP Concentration

The molar ratio of PAP-to-mRNA used in the polyadenylation experiments was 1:15 to 1:150. *E. coli* PAP is not sequence-specific; it recognizes 3′ end of mRNA and can also bind to poly(A) sequence [40]. PAP is fully distributive as an isolated enzyme, therefore the rate of mRNA tail growth depends on PAP concentration (reviewed in [41]). Indeed, the concentration of PAP enzyme was found to positively influence ATP consumption, demonstrating faster reaction kinetics at increased PAP enzyme concentration (Figure 2a–c). Longer poly(A) tails will be produced in the same time interval when higher PAP concentrations are used (Figure 2b). Although PAP concentration was not explicitly included as a parameter in the simple model we used, its effect can be seen from increased reaction rate k with PAP concentration (Figure 2c).

ATP half-life ($t_{1/2}=ln\left(2\right)/k$) was different for the three curves shown. At lower PAP concentrations, $t_{1/2}\left(1\;\mathrm{ng}/\mu L\;\mathrm{PAP}\right)\approx 71$ min, while for higher concentrations, $t_{1/2}\left(5\;\mathrm{ng}/\mu L\;\mathrm{PAP}\right)\approx 13$ min and $t_{1/2}\left(10\;\mathrm{ng}/\mu L\;\mathrm{PAP}\right)\approx 7$ min. Consequently, if the reaction duration is much longer than the half-life, all ATP will be consumed, resulting in the longest possible tail length. These results are confirmed in Figure 2a: the reaction time of 1 hr is long enough for two larger concentrations of PAP (5 and 10 ng/µg), but not for the lowest one (1 ng/µg).

The maximal tail length, $N_{tail}\approx C_{ATP}\left(0\right)/C_{mRNA}$ (see Equation (7a)), should be same for all three curves, since both the initial concentration of ATP and the mRNA concentration are constant. Slight deviations in $N_{tail}$ values shown in Figure 2b reflect the experimental realisation.

At all PAP concentrations, polyadenylated mRNA purified by Oligo dT (ligand with high selectivity for poly(A) tails) showed >95% recovery in elution, suggesting that >95% of all mRNA molecules were polyadenylated. This indirectly demonstrates the distributive nature of PAP, which polyadenylated >95% of all mRNA molecules in solution despite the low molar ratio of PAP to mRNA (1:15 to 1:150).

### 2.3. Impact of Initial ATP Concentration

The initial concentration of ATP ($x_{0}$) is explicitly entering our model as a parameter (Equation (6)). For the intermediate values (5 ng/μL) of the PAP concentration, the reaction was completed within 60 min (Figure 2d), reaching the predicted maximal possible tail lengths (Figure 2e, dotted lines) for all three cases considered. For the mDeco used (molecular weight: 381,000 g/mol), the maximal tail lengths can be estimated using Equation (7a) as 749 nt (1.0 mM ATP, 509 ng/μL mRNA), 498 nt (0.7 mM ATP, 536 ng/μL mRNA) and 317 nt (0.4 mM ATP, 481 ng/μL mRNA). The reaction rate constant *k*, according to Equation (9), should be inversely proportional to initial concentrations of ATP. of the data in Figure 2f are well fit by $f\left(x_{0}\right)=0.043/x_{0}$, in excellent agreement with our model (Equation (9)). This inverse dependence implies higher reaction rate constants at lower initial ATP concentrations. In other words, at same PAP concentrations, higher ATP concentrations require more time to be fully consumed.

### 2.4. Impact of mRNA Concentration

Similarly, varying mRNA concentrations affected ATP consumption (Figure 2g), namely, the increase of mRNA concentration resulted in faster reaction kinetics and larger reaction rate constants. Highest tested mRNA concentration (grey) had a half-life of 38 min, and during 60 min of observation almost reached the targeted tail length of 405 nt. Lowering concentration of mRNA 2-fold (orange) resulted in half-life of 76 min; 60 min reaction time was therefore insufficient to reach the maximal tail length of 731 nt. Half-life for 4-fold lower mRNA concentration (blue) has a half-life of 268 min and could not reach the theoretical maximal tail length of 1581 nt. Due to the small relative concentration of PAP used (1.5 ng per μg of mRNA) none of the reactions reached completion during the 60 min of observation time (Figure 2g,h), but that does not affect the qualitative conclusions. Since the same amount of PAP was used, the difference in reaction velocity seemed surprising at first: the amount of mRNA:PAP complexes, which serve as reaction centres, should have been the same. However, due to the distributive character of PAP enzymes, PAP can adsorb and desorb to multiple mRNA molecules before a productive integration of ATP into an mRNA tail. Therefore, increased number of mRNAs provides a larger number of potential reaction centres. Chemically, this can be qualitatively justified as follows: the activation energy of adsorption is at least an order of magnitude lower than the activation energy of PAP reaction. In view of Arrhenius equation for the reaction rate constant $k=Ae^{-E_{a}/RT}$, where A is the pre-exponential factor, $E_{a}$ is the activation energy, R is the universal gas constant and T is temperature, the higher the activation energy, the lower the value of the reaction rate constant at other equal conditions. Since half-life is inversely proportional to the reaction rate constant $t_{1/2}=ln\left(2\right)/k$, adsorption/desorption is faster than polyadenylation. PAP therefore undergoes frequent intermolecular transfer between mRNA substrates, whereas de novo initiation of poly(A)-tail synthesis occurs only rarely. Overall, our results can explain the approximately linear increase of the reaction rate constant with mRNA concentration (Figure 2i).

Optimal reaction conditions to reach a tail length of ~200 nt within 60 min is 500–1000 ng/μL of mRNA and 5–10 ng/μg of PAP. The exact value of the tail length can be tuned with the starting ATP concentration using Equation (7a), as explained above.

### 2.5. Development of Poly(A) Tail Length Determination Method with IP-RPC

**Figure 3:**
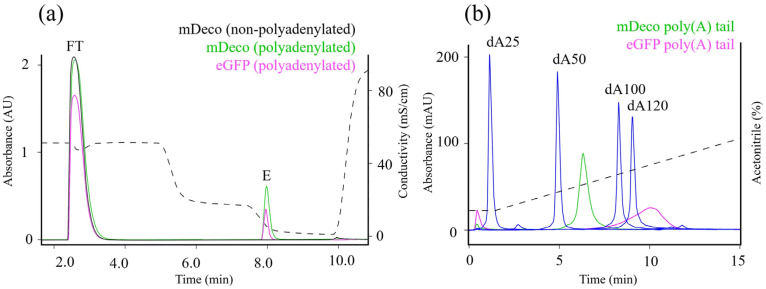
Poly(A) tails isolated with Oligo dT column and subsequently separated by chain length on CIMac SDVB column. (**a**) Following T1 cleavage, digestion products were removed in Oligo dT flow-through (FT), while poly(A) tail bouns to Oligo dT and was eluted (E) in water. mRNAs polyadenylated with PAP bound to Oligo dT column, while non-polyadenylated mRNA did not bind. (**b**) After Oligo dT purification, poly(A) tails were resolved with IP-RPC analytical chromatography using CIMac SDVB and their retention times compared to oligo-deoxyadenine standards (blue trace). Dashed line in (**a**) indicates conductivity and dashed line in (**b**) indicates increasing acetonitrile gradient.

To validate our approach to control mRNA polyadenylation via ATP consumption, we then developed IP-RP LC method based on CIMac SDVB monolith matrix. The method depends on cleavage of poly(A) tail with a selective RNAse, followed by IP-RP LC separation to determine the molecular size of the cleaved fragment by comparison to a synthetic standard. Polyadenylated samples were digested with RNase T1, which cleaves a single-stranded RNA chain at guanosine residues, therefore leaving the poly(A) tail intact. T1 was used to digest non-polyadenylated mRNA (mDeco, negative control), sequence-encoded polyadenylated mRNA (eGFP, positive control) and enzymatically polyadenylated mRNA (mDeco, test sample). After digestion, all samples were purified with Oligo dT chromatography to remove T1 digestion products in flow-through, while poly(A) tail bound to Oligo dT in the presence of 0.5 M NaCl and was eluted in water. No binding to Oligo dT was observed for non-polyadenylated mRNA (Figure 3a), indicating that there was no non-specific binding or longer adenine-rich stretches present in mDeco sequence (Oligo dT binds adenine sequence > 10 nt in length [42]). PAP-polyadenylated mDeco and sequence-encoded poly(A) tail mRNA (eGFP) both showed binding to Oligo dT (Figure 3a). The Oligo dT eluates containing poly(A) tails were initially separated with CIMac SDVB column in ascending acetonitrile gradient with triethylammonium acetate (TEAA) as ion pairing reagent, and compared to retention times of poly-deoxyadenosine synthetic standards of defined lengths (dA25, dA50, dA100 and dA120) (Figure 3b). Poly(A) species of plasmid-encoded poly(A) mRNA (eGFP) eluted between dA50 and dA100 in agreement with the expected length of 65 nucleotides. Poly(A) species of mDeco mRNA eluted after dA120, consistent with prediction of poly(A) length determined theoretically based on ATP consumption. As shown previously [43,44] a difference in poly(A) peak shape was observed between enzymatically added poly(A) and sequence-encoded poly(A).

**Figure 4:**
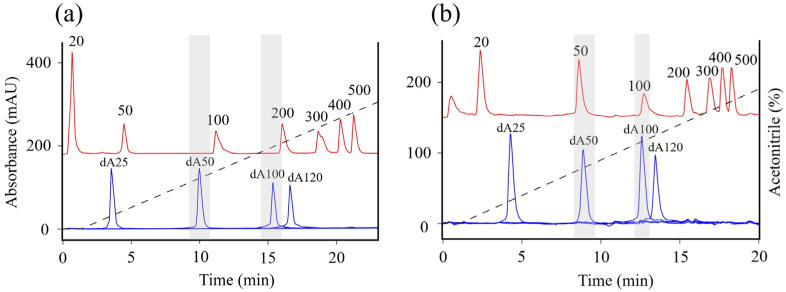
Analytical PATfix chromatograms (UV 260 nm) of DNA oligo-adenines (blue trace) and RNA Marker Low (red trace) separated with CIMac SDVB with mobile phase containing (**a**) TEAA or (**b**) DBAA. Gray bands represent elution of 50 and 100 nt DNA oligo-adenines compared to elution of 50 and 100 nt fragments of RNA Marker Low standard mixture. Dashed lines indicate increasing acetonitrile gradient.

To determine poly(A) tail lengths >120 nt, longer-chain analytical standards were required. The longest commercially available oligo-adenine standard is 120 nt RNA; we therefore used ‘Marker Low’ RNA standard containing RNA synthetic standard from 20 to 500 nt. But when comparing the retention times of oligo-adenines and RNA Marker Low using TEAA as an ion pairing reagent, retention time alignment of similar lengths was poor (Figure 4a).

We replaced TEAA with a more hydrophobic ion-pairing reagent, dibutylammonium acetate (DBAA) [45], which showed significant improvement in the retention time alignment of oligo-adenines and RNA Marker Low (Figure 4b).

Additionally, a polynomial regression generated from RNA standard marker retention times was used to predict the length of oligo-adenine standard (dA25–dA120) retention times; predicted and measured dA lengths matched within ± 5%. Next, peak retention time conversion to RNA chain length based on RNA Marker Low retention time was applied to determine poly(A) tail lengths in mRNA samples.

**Figure 5:**
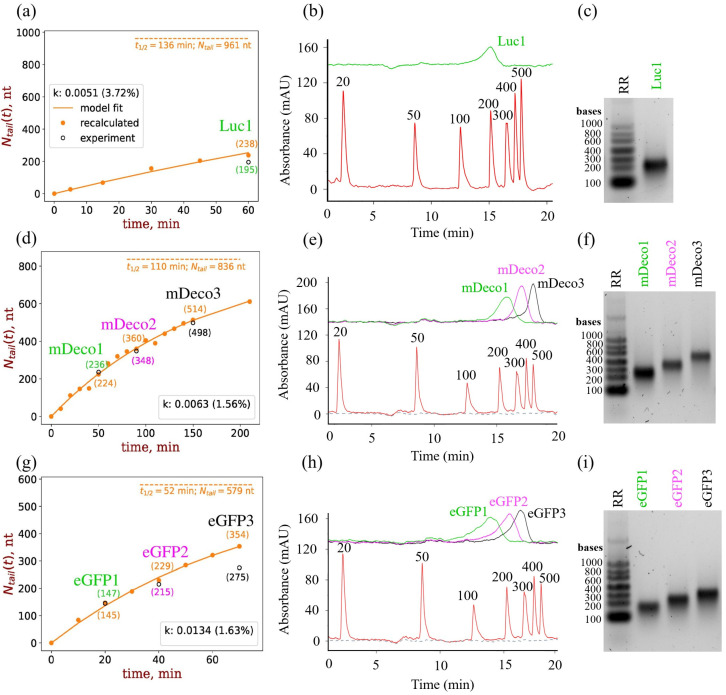
Measured vs. calculated tails lengths for three mRNA constructs: Luciferase mRNA (1711 nt), mDeco mRNA (1260 nt) and eGFP mRNA (915 nt) at polyadenylation conditions of 500 ng/μL mRNA, 1 mM ATP, 1.5 ng/μg PAP. First column (**a**,**d**,**g**) shows time dependencies of tail lengths for different mRNAs. Equation (6) is used to fit ATP concentration data (not shown) and obtain reaction rate constant k (see legends); tail lengths are recalculated point by point (filled dots) or redrawn using the fit (solid line) using Equation (7); the tail length values, measured with CIMac SDVB method (hollow dots) and recalculated using Equation (7) are shown in brackets for comparison. Horizontal dashed line is the visualisation of the steady state (*t* >> *t*_1/2_ = *ln*(2)/*k*) limit value for the tail length (value estimated from Equation (7a) shown under the line). (**b**,**e**,**h**) Analytical chromatograms of (CIMac SDVB) of poly(A) tail lengths; red trace corresponds to RNA Marker low (Abnova, Taipei, Taiwan). (**c**,**f**,**i**) are agarose gel electrophorogramsof the same poly(A) tails. RR:RiboRuler Low Range RNA Ladder (Thermo Scientific, Waltham, MA, USA).

Three mRNA constructs of different lengths (mDeco, eGFP, Luc) were used to validate the SDVB analytical method on a set of samples with different poly(A) lengths. mRNAs were PAP-polyadenylated, reaction was monitored for ATP consumption in 10 min intervals. Reaction was quenched after ~200 µM, 400 µM and 600 µM of ATP was consumed and reaction product purified with Oligo dT column. Poly(A) tail lengths were determined using CIMac SDVB method as described above (Figure 5b,e,h) as well as with agarose gel electrophoresis (Figure 5c,f,i). Results were compared with theoretically calculated poly(A) lengths based on ATP consumption, using Equation (7). Values were in close agreement, with 2–22% difference in length estimation between SDVB-determined and theoretically calculated poly(A) tail (Table 1).

Results of time-dependent measurements, together with fits using Equation (6), are shown in Figure 5a,d,g. The values of reaction rate coefficients (with fit errors in brackets) are shown in the legends. Figure 5a,d,g, show that the model adequately describes the PAP reaction, preserving the stoichiometry, and agrees with the experimentally confirmed values of the poly(A) lengths to a very high degree.

Finally, mDeco mRNA with three distinct poly(A) tail lengths was tested in cellular models. Vaccinia capping enzyme (VCE)-capped mRNAs were differentially polyadenylated by stopping PAP reaction at three time points. The polyadenylated mDeco mRNAs were purified using Oligo dT, followed by IP-RPC (SDVB) purification, and subsequently buffer-exchanged into ddH_2_O. The final mRNA samples were subjected to dot blot analysis, which confirmed significant reduction in dsRNA signal between IVT and purified sample (Figure S2), rendering them suitable for cellular assays. The length of poly(A) tails was assessed using CIMac SDVB method, showing poly(A) tail lengths of 324 nt, 529 nt, and 702 nt (for simplicity in figures, poly(A) tail lengths are referred as ‘300 nt’, ‘500 nt’, and ‘700 nt’, respectively).

### 2.6. Poly(A) Length Effect on Decorator Expression

**Figure 6:**
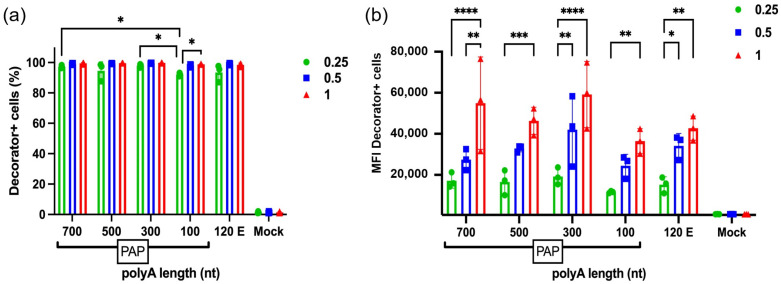
Expression profile of mDeco mRNA with different poly(A) lengths at 0.25, 0.5 and 1 µg/well. Flow cytometry data shows positive A549 cells (**a**) and MFI transfections for the respective conditions (**b**). This dose-response study demonstrates that both the mRNA input dose and poly(A) tail length synergistically affect protein expression as protein output scales with input mRNA. Higher-poly(A) length mRNA resulted in higher protein expression compared to the shorter poly(A) mRNAs (100 nt or 120 E (E is abbreviated as template-encoded poly(A) tail)). Data are shown as mean ± SEM (*n* = 3 biologically independent samples). Two-way ANOVA with Tukey’s multiple comparison test was applied, where * *p* < 0.05; ** *p* < 0.01; *** *p* < 0.001; **** *p* < 0.0001.

We explored the cellular effects of the poly(A) tail length of mDeco in vitro. Low (0.25 μg/well), medium (0.5 µg/well) and high dose (1 μg/well) of these mRNAs were transfected on A549 cells. After 24 h, almost 92–99% of cells were expressing Decorator (Figure 6a) at all dosing amounts. The highest mRNA dose (1 µg) produced higher protein expression than 0.5 µg or 0.25 µg. The mean fluorescence intensities (MFI) for all conditions were dose-dependent for the respective poly(A) lengths. Enzymatically tailed mRNAs with longer poly(A) lengths (≥300 nt) consistently resulted in higher and more sustained protein expression across a range of doses. For all doses, higher length tails had higher MFI compared to the 120 encoded (120 nt) or shorter enzymatic (100 nt) poly(A) tail. Overall, the poly(A) length of 300 nt performed better than the 700 nt and 500 nt tails (Figure 6b). We also tested the immunogenicity of these mRNAs and found that they did not elicit the IFNA1 or TNF gene; however, we noticed a slight increase in the IL6 gene expression for 700 nt, 500 nt and 300 nt tailed mRNAs compared to the mock-treated cells (Figure S3). This suggested that the PAP reaction did not trigger any dramatic RNA-mediated immune response genes. These findings underline the tolerance and utility of longer-poly(A)-tailed mRNA, where dose minimization is desired for in vivo delivery or cost-limited applications.

### 2.7. Optimized Poly(A) Length of mRNA Enhances mDeco Expression

**Figure 7:**
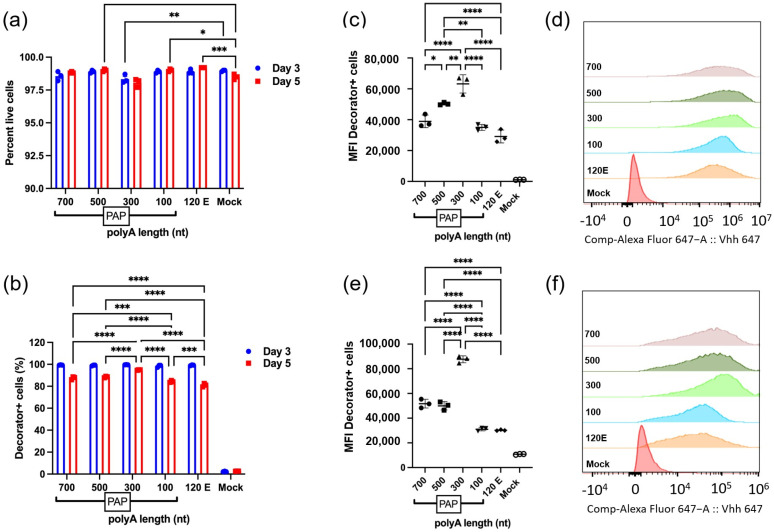
Flow cytometry-based expression profile of mDeco mRNA with different poly(A) lengths at 0.5 µg/well at days 3 and 5 after transfection. The percent of live cells (**a**) and mDeco-positive cells (stained with Alexa Flour 647) (**b**) show distinct MFI (**c**), and MFI shifts at days 3 (**d**) and 5 (**e**,**f**). We observed that the longer tail length mRNAs outperformed the shorter mRNAs with shorter poly(A) tails (for both, enzymatic or 120 E (E is abbreviated as template-encoded poly(A) tail)). Data are shown as mean ± SEM (*n* = 3 biologically independent samples). Two-way ANOVA with Sidak’s multiple comparison test was applied for (**a**,**b**). One-way ANOVA with Tukey’s multiple comparison test was applied for (**c**,**e**). *p* values were indicated as * *p* < 0.05; ** *p* < 0.01; *** *p* < 0.001; **** *p* < 0.0001.

To understand the optimal length of mRNA that gives extended protein expression, we performed flow cytometry on days 3 and 5 (0.5 µg/well). Almost all the cells were mDeco-positive on day 3; however, on day 5 it reduced, ranging between 75–90% for all lengths of mRNAs (Figure 7b). At day 3, mRNA species with longer poly(A) tails showed markedly higher protein expression compared to those with shorter tails (100 nt) and the 120 nt template-encoded mRNA. The same trend was observed for day 5, mRNAs with longer tails maintained higher expression, indicating greater durability and translation longevity. There was a decline in protein expression from day 3 to day 5 across all mRNA constructs, but the decrease was more pronounced for shorter tails (100, 120 E). Although the intensities were decreased at day 5 for all mRNAs, it was clear that the MFI spread and shift for the 100 nt and 120 E was distinctly lower and more spread out compared to the longer poly(A) tailed mRNAs (Figure 7d,f). It was interesting to observe that the 300 nt tail mRNA resulted in consistently higher MFI values at day 3 and 5 (Figure 7c,e) compared to 700 nt and 500 nt poly(A) tailed mRNAs. It may be possible that the cells receiving the exogenous mRNA regulate tail length and tolerate a certain maximum tail length. Even with this anomaly our results collectively show that enzymatically added poly(A) tails of ≥300 nt significantly enhance protein expression and mRNA persistence in mammalian cells compared to shorter or template-encoded tails.

Our findings align with the established understanding that in mammalian cells, longer poly(A) tails recruit more cytoplasmic poly(A)-binding protein (PABPC), which promotes closed-loop mRNA structure, protects against deadenylation, and enhances translation initiation and ribosome recycling [46]. However, it seems that mRNA decay is predominantly initiated by deadenylation, with initial tail length setting a buffer period before decay begins after achieving a length of 250 nt [47]. Thus, our findings align with the findings that increased tail length prolongs the functional lifespan of IVT mRNAs, especially when enzymatically controlled to achieve longer tailing.There are some correlation reports about this relationship based on the transcriptome studies of steady-state somatic mammalian cells. For native mRNAs, PABPC interactions with initiation factors (eIF4E/eIF4G) correlate strongly with mRNA stability and translation more than the length of the poly(A) tail alone. Furthermore, the UTRs may contain microRNA sites that modulate the stability and expression of the mRNA [48]. However, such studies reflect endogenous regulation in steady state and do not fully account for exogenously delivered IVT mRNAs where tail length can vary widely and lack associated regulatory cis-elements. Our findings are consistent with the hypothesis that by increasing the tail length of IVT mRNA beyond typical endogenous ranges via PAP processing, the window before deadenylation-dependent decay is effectively extended and protein output maximized.

## 3. Materials and Methods

### 3.1. Template DNA Preparation

The gene inserts were synthesized and cloned into Twist High Amp (Twist Biosciences, San Francisco, CA, USA) plasmid vector. Glycerol stocks for positive clones were expanded in LB broth, and plasmid was extracted using Qiagen mini/midi plasmid kit (Qiagen, Hilden, Germany) as instructed by the manufacturer. Plasmid DNA was linearized with Not-I HF (New England Biolabs, Ipswich, MA, USA) per the recommended protocol. Linearized plasmid was purified using the QIAquick PCR Purification Kit (QIAGEN GmbH, Hilden, Germany) or sodium acetate–ethanol method. The final purified linear DNA was further used for the IVT reaction.

### 3.2. IVT Reaction

RNase inhibitor (40 U/µL stock concentration), Pyrophosphatase (100 U/mL stock concentration), T7 RNA polymerase (50 U/µL stock concentration), ATP, CTP and GTP (100 mM stock concentration) were from Mebep Bioscience, Shenzhen, China, and N1-methylΨ UTP (100 mM stock concentration) was from Trilink Biotechnologies, San Diego, CA, USA. We obtained 1 M MgCl_2_ from Invitrogen, Waltham, MA, USA, and 10x IVT buffer (400 mM Tris; 20 mM spermidine; 10 mM DTT; pH 7.9) was prepared in house. The IVT mixture was prepared as described in Table 2 and incubated in Thermomixer™ C (Eppendorf, Hamburg, Germany) at 37 °C and 300 rpm. mRNA production and NTP consumption were monitored by CIMac PrimaS^®^ (Sartorius BIA Separations, Ajdovščina, Slovenia) PATfix^®^ analytics. After the mRNA production in IVT reached the plateau, the reaction was inactivated with a final concentration of 50 mM EDTA, pH 8.

### 3.3. Multimodal Chromatography Purification of mRNA

Quenched IVT containing non-polyadenylated mRNA was purified with Swiper multimodal chromatography, as described before [49]. Purification was done on the ÄKTA pure™ 150 (Cytiva, Uppsala, Sweden) FPLC system, and Unicorn software (version 7.9.0, Cytiva, Uppsala, Sweden) was used for instrument control and data acquisition. The quenched IVT reaction was 10-fold diluted in 50 mM citrate (pH 5) and loaded onto the column. The majority of IVT reaction components were removed in the flow-through, while linear plasmid was eliminated by high salt wash with 50 mM citrate and 0.45 M NaCl at pH 5.0. mRNA was eluted in a pH gradient achieved by a step into 100 mM Na-phosphate at pH 7.5. After Swiper purification, samples were buffer-exchanged into ddH_2_O on 30 kDa VivaspinTurbo-4 spin columns (Sartorius, Göttingen, Germany).

### 3.4. Capping Reaction with VCE

For mRNA used in cellular activity studies and, where indicated, for PAP optimization studies, mRNA was capped with the vaccinia capping enzyme (VCE) system. Purified mRNA was first denatured at 65 °C for 10 min in Thermomixer™ C (Eppendorf, Hamburg, Germany) at 37 °C. Denatured mRNA was cooled down in a benchtop cooler. GTP (100 mM stock concentration) was from Mebep Bioscience, Shenzhen, China, and S-adenosylmethionine (SAM) (4 mM stock concentration) was from New England Biolabs, Ipswich, MA, USA. Enzymes, Guanylyltransferase (2 mg/mL stock concentration), 2′-O-Methyltransferase (1.8 mg/mL stock concentration) and RNase Inhibitor (1.5 mg/mL stock concentration) were from Aldevron, Fargo, ND, USA. A 10x Capping buffer (500 mM Tris; 50 mM KCl; 10 mM MgCl_2_; 10 mM DTT; pH 8) was prepared in house. Post-transcriptional capping reaction was prepared as described in Table 3 and incubated at 37 °C and 300 rpm for 1 h. After capping reaction, mRNA was immediately used for polyadenylation.

### 3.5. Polyadenylation Reaction

ATP (100 mM stock concentration) and 10x Polyadenylation buffer (500 mM Tris; 2.5 M NaCl; 100 mM MgCl_2_; pH 7) used was from Mebep Bioscience, China. *E. coli*-derived poly(A) Polymerase (3 mg/mL stock concentration) was from Aldevron, Fargo, ND, USA. Unless stated otherwise, 1x Polyadenylation buffer, 1 mM of ATP, 0.5 mg/mL mRNA and 1.5 ng/μg of PAP enzyme was used in the polyadenylation reaction. Reactions were incubated in Thermomixer™ C (Eppendorf, Hamburg, Germany) at 37 °C and 300 rpm. 5 μL aliquots were taken out of the PAP reaction at defined timepoints and quenched with a 50 mM EDTA final concentration. Samples were analyzed with HPLC analytics using a CIMac PrimaS^®^ 0.1 mL Analytical column (Sartorius BIA Separations, Ajdovščina, Slovenia).

### 3.6. mRNA Purification with Oligo dT Affinity Chromatography

Chromatographic purification of polyadenylated mRNA was performed on ÄKTA pure™ 150 (Cytiva, Uppsala, Sweden) FPLC system composed of two pumps and a multi-wavelength UV-Vis detector (2 mm flow cell path length). Unicorn software (Cytiva, Uppsala, Sweden) was used for instrument control and data acquisition. The polyadenylation reaction mixture was diluted 10-fold in loading buffer (50 mM sodium phosphate, 0.5 M NaCl, pH 7.4) and loaded onto a CIMmultus^®^ Oligo dT18 (2 µm channel size) column (Sartorius BIA Separations, Ajdovščina, Slovenia). After unbound enzymatic reaction components eluted in the flow-through and the UV 260 nm signal were stabilized, a wash step was performed with 50 mM sodium phosphate, 0.1 M NaCl, and pH 7.4, followed by the step elution of polyadenylated mRNA with ddH_2_O.

### 3.7. mRNA Polishing with Reverse-Phase Ion-Pair (IP-RP) Chromatography

mDeco samples which were used for cellular tests were additionally purified with a CIMmultus^®^ SDVB (IP-RP) column with 2 µm channels (Sartorius BIA Separations, Ajdovščina. Slovenia). Purification was performed with ÄKTA pure™ 150 (Cytiva, Uppsala, Sweden) FPLC system composed of two pumps and a multi-wavelength UV-Vis detector (2 mm flow cell path length). Unicorn software (Cytiva, Uppsala, Sweden) was used for instrument control and data acquisition. Oligo dT eluate was first diluted 10-fold into loading buffer (50 mM TEAA, 7.5% acetonitrile, pH 7.0) and loaded onto the column at 3 CV/min (CV; column volume). Elution with 50 mM TEAA, 18% acetonitrile pH 7.9 was subsequently performed in a 95 CV long linear gradient at 2 CV/min. Elution fractions that were collected during linear gradient were immediately buffer-exchanged into ddH_2_O and analyzed for presence of dsRNA on J2 dot blot. Fractions that did not exhibit dsRNA signal were pooled together and buffer-exchanged into ddH_2_O with 30 kDa VivaspinTurbo-4 spin columns (Sartorius, Gottingen, Germany) and used in cellular tests.

### 3.8. Analytics

#### 3.8.1. CIMac PrimaS Analysis for Determination of ATP Consumption

ATP concentration in the PAP reactions was determined on a PATfix^®^ LC system with a CIMac PrimaS^®^ 0.1 mL analytical column (2 µm channel size), which was used for separation of ATP from mRNA. PATfix^®^ 2.0 software (Sartorius BIA Separations, Ajdovščina, Slovenia) was used for instrument control, data acquisition and data analysis. EDTA-quenched samples from PAP reactions were diluted in mobile phase A (MPA; 50 mM HEPES, pH 7.0). Injection volume was 25 µL andflowrate 2 mL/min. ATP from PAP reaction was separated with a linear gradient from 0 to 45% of mobile phase B (MPB; 50 mM HEPES, 100 mM sodium pyrophosphate, pH 8.3) and 55% of MPA over 20 CV. Next, an isocratic step of 55% MPA and 45% of MPB for 14 CVs was followed by a step to 40% MPB and 60% mobile phase C (MPC; 0.1 M NaOH, 1 M NaCl) for an additional 14 CVs to elute mRNA. Column was regenerated with 18 CVs of 100% MPC and equilibrated with 2 CV of mobile phase D (MPD; 0.5 M HEPES, pH 7.0) and 90 CV of MPA. ATP standard calibration curve was prepared by serially diluting a 100 mM stock ATP Solution (Mebep Bioscience, Shenzhen, China) to a final concentration range of 2–20 µM. ATP calibration curve was used to calculate ATP consumption and therefore poly(A) tail length. Concentration of ATP at different timepoints during PAP reaction was calculated using the following equation:

$$c\left(ATP\right)=\frac{\left(A\left(A260\right)-n\right)}{k}*Dil.factor$$

*c*(*ATP*) = concentration of ATP in sample;
*A*(*A*260) = peak area at absorbance of 260 nm;
*k* = slope of standard curve for ATP;
*n* = y-intercept of standard curve for ATP;
*Dil.factor* = dilution factor.

#### 3.8.2. RNase T1 Digestion and Purification of Poly(A) Tail

RNase T1 digestion was done as previously described 30. An amount of 50 µg or 100 µg of polyadenylated mRNA was digested using RNase T1 (1000 U/µL stock concentration) at a concentration of 40 U of RNase T1 per 1 µg of mRNA and 1x rCutSmart buffer, both supplied by New England Biolabs, Ipswich, USA. The reaction was incubated at 37 °C for 30 min and 300 rpm in Thermomixer™ C (Eppendorf, Hamburg, Germany). After T1 digestion, poly(A) tails were purified using Oligo dT affinity chromatography under the same conditions as described in Materials and Methods. Purified poly(A) tails were analyzed on agarose gel electrophoresis and with CIMac SDVB analytics.

#### 3.8.3. IP-RP Analytical Chromatography with UV Detection

Reversed-phase chromatography was performed using the PATfix^®^ LC system and a CIMac SDVB 0.3 mL (2 μm) analytical column at an elevated temperature of 60 °C. The UV absorbance signal was measured at 260 nm. Data analysis was carried out using PATfix 2.0 software. Oligo-deoxyadenylic acids (DNA oligo-adenines) of four distinct lengths (25, 50, 100, and 120 nucleotides), from Eurofins Genomics, Germany, and RNA Marker Low, obtained from Abnova, Taiwan, were used as standards for the method development and poly(A) tail length determination. Poly(A) samples, purified as described above, as well as DNA oligo-adenines were diluted to a concentration of 1 ng/μL, and RNA Marker Low was diluted 100-fold. The injection volume was 250 μL. For SDVB analysis, all samples were diluted in either TEAA or DBAA with a low acetonitrile content. After sample injection, the investigated species were eluted using an increasing linear gradient of acetonitrile.

### 3.9. Agarose Gel Electrophoresis

Agarose gel electrophoresis was used for visualization of mRNA and poly(A) tail lengths. mRNA and poly(A) tail samples were diluted to 4 ng/μL in ddH_2_O, 18 μL of samples were then mixed with 2 μL of TriTrack loading dye (Thermo Fischer Scientific, Waltham, MA, USA) and loaded onto 1.2% agarose gel. Electrophoresis was performed at 100 V for 90 min in 1x TAE (40 mM Tris-acetate, 1 mM EDTA, pH 8.3) as a running buffer. Gel was stained with SybrGold (Invitrogen, Carlsbad, CA, USA) for 30 min and visualized with iBright (Thermo Fischer Scientific, Waltham, MA, USA).

### 3.10. J2 Dot Blot Analysis

mRNA samples were analyzed on J2 immunoblots for dsRNA detection as previously reported [50].

### 3.11. Cell Expression of mRNA

Stability and expression of mRNA in vitro was tested using DecoratorTM (mDeco), a proprietary reporter mRNA with PAP-controlled poly(A) tails of 700 nt, 500 nt, and 300 nt or encoded 120 nt (120 E). mDeco is a specialized mRNA reporter designed for the easy screening of transfecting reagents, polymers and lipid nanoparticles (LNPs). The cells receiving mDeco mRNA express multiple commonly used tags (V5, HA, c-myc and His tag) and a VHH (which is non-reactive to mammalian, bacterial and viral proteins) on their cell surfaces. We first tested the expression of mDeco mRNA via microscopy and flow cytometry.

### 3.12. Immunofluorescence Microscopy

Briefly, A549 cells (125,000) were plated in 24 well black plate in DMEM media supplemented with 10% fetal bovine serum and 1% penicillin–streptomycin and incubated at 37 °C with 5% CO_2_. The next day, cells were transfected with 1 µg mRNA per well using Lipofectamine MessengerMAX reagent (Life Technologies, Carlsbad, CA, USA). After 24 h, cells were fixed with 4% paraformaldehyde and stained with 1:250 anti-VHH Alexa Fluor 647 (Genscript, Piscataway, NJ, USA), and unlabeled primary antibodies for V5, HA, myc and His tag (Cell Signaling Technology, Danvers, MA, USA) were further stained with Alexa fluor-labeled antibodies. Staining was performed at 37 °C for 30 min of incubation. Nuclei were stained with DAPI, and cover slips were placed after prolonged treatment. Images were acquired using an EVOS M7000 fluorescence microscope (Thermo Fisher Scientific, Waltham, MA, USA).

### 3.13. Flow Cytometry

After tranfection with mDeco (0.25 or 0.5 or 1 µg) using the MessengerMAX reagent as described above, cells were trypsinized on day 1, 3 or 5. Cells were collected and stained for viability using the LIVE/DEAD Fixable Aqua Dead Cell Stain kit (Life technologies, Carlsbad, CA, USA). Cells were then stained for V5, HA, myc tags and/or VHH as described above and incubated on ice for 30 min. Cells were washed with PBS and then analyzed using a NorthernLights flow cytometry (Cytek, Fremont, CA, USA) with live spectral unmixing. Data was collected and analyzed using FlowJo software (version 10.10, BD Biosciences, San Jose, CA, USA).

### 3.14. Immunogenicity

Cellular effects of the poly(A) tail lengths (enzymatically added 300, 500 and 700 nt tails) or encoded 120 nt mDeco mRNA were tested after 24 h of transfection. Total mRNA was extracted from cells using the RNeasy Plus kit (Qiagen, Hilden, Germany). The RNA amount was quantified with Nanodrop (Thermo Fisher Scientific, Waltham, MA, USA), and cDNA was prepared using the High capacity cDNA Reverse Transcription kit (Applied Biosystems, Waltham, MA, USA). Genes expressing cytokines were assessed with Fast Advanced TaqMan master mix (Applied Biosystems, USA) and primer–probe sets for IFNA1 (Hs00855471_g1), TNF (Hs00174128_m1) and IL6 (Hs00174131_m1). Relative expression compared to controls was measured with QuantStudio6 Pro (Applied Biosystems, Carlsbad, CA, USA).

## 4. Conclusions

The DNA template used for mRNA production in the IVT reaction is the most frequently produced in microbial host cells, such as *E. coli*.

Microbial production of pDNA encoding long poly(A) tails is challenging and leads to multimerization of pDNA, sequence instability, limited control over tail length, and cloning constraints that restrict flexibility in mRNA design. This can be mitigated by producing mRNA with post-transcriptional enzymatic polyadenylation using PAP. In this study we report the first successful monitoring of ATP consumption to control PAP activity for mRNA production. We use ATP consumption as a process control metric for PAP-based polyadenylation. By modeling ATP utilization using PAP enzyme, substrate, and mRNA concentration as variables, we achieved near-real-time and accurate poly(A) tail length determination, validated by a newly developed IP-RP LC method using on analytical monolith column. The two methods resulted in 2–22% difference between measured and theoretically calculated poly(A) tail lengths.

We produced multiple mRNAs with controlled poly(A) tails of 300–700 nt and demonstrated that mRNAs with longer poly(A) tails showed higher and extended expression of reporter mRNAs, compared to the shorter tails which were either added enzymatically or co-transcriptionally. The uptake and expression of longer poly(A) tail lengths did not significantly alter the cytokine profile, showing that mRNAs with longer poly(A) tails can be reliably synthesized and are desirable for better expression.

To the best of our knowledge, this is the first report on the use of ATP monitoring to control polyadenylation of mRNA, supporting a scalable, sequence-independent approach for mRNA manufacturing and enabling precise and reproducible polyadenylation tailored to therapeutic specifications.

Furthermore, due to the dependence of many biosynthetic conversions on ATP, our approach paves the way to study and control other ATP-dependent enzymatic reactions, some with significant industrial potential.

## Figures and Tables

**Table 1:** Comparison of poly(A) tail lengths of PAP-polyadenylated mRNAsdetermined by AGE, CIMac SDVB and theoretical prediction based on ATP consumption (measured by CIMac PrimaS). Relative differences between CIMac SDVB-determined and ATP-consumption derived values are reported.

Analytical Method	mDeco1	mDeco2	mDeco3	eGFP1	eGFP2	eGFP3	Luc1
**AGE (nt)**	200–300	300–400	400–600	100–200	200–300	300–400	200–300
**CIMac SDVB (nt)**	236	348	498	145	215	275	195
**Via [ATP]/Equation (7) (nt)**	224	359	513	147	229	353	238
**% difference SDVB/PrimaS**	5	3	4	2	6	22	18

**Table 2:** IVT reaction mixture.

MgCl_2_ (mM)	NTP (mM)	DNA (ng/µL)	T7 RNAP (U/µL)	RNAse Inhibitor (U/µL)	Pyrophosphatase (U/mL)
32	10	40	5	1	1

**Table 3:** VCE reaction mixture.

RNase Inhibitor (µg/mL)	GTP (mM)	mRNA (mg/mL)	SAM (mM)	Guanylyltransferase (µg/mL)	2′-O-Methyltransferase (µg/mL)
5	1	1	0.5	5	50

## Data Availability

The original contributions presented in this study are included in the article/Supplementary Material. Further inquiries can be directed to the corresponding authors.

## Supplementary Materials

The following supporting information can be downloaded at: https://www.mdpi.com/article/10.3390/ijms27072928/s1. References [51,52,53,54] are cited in Supplementary Materials.

## Abbreviations

The following abbreviations are used in this manuscript:

ATP	Adenosine triphosphate
CV	Column volume
DBAA	Dibutylammonium acetate
GFP	Green fluorescent protein
IP-RPC	Ion pair reverse-phase chromatography
IVT	In vitro transcription
LC	Liquid chromatography
Luc	Luciferase
mDeco	Decorator mRNA
nt	Nucleotide
NTP	Nucleoside triphosphate
PABPs	Poly(A)-binding proteins
PAP	Poly(A) polymerase
SDVB	Styrene divinyl benzene
TEAA	Triethylammonium acetate
VCE	Vaccinia capping enzyme
VHH	Variable heavy chain domain of a heavy chain antibody
