# Supplementary material for: Controlling In Vitro mRNA Polyadenylation by Monitoring Poly(A) Polymerase Consumption of ATP
Source: Int J Mol Sci. 2026 Mar 24;27(7):2928. doi: 10.3390/ijms27072928 (PMC13073625; doi:10.3390/ijms27072928)
Supplement: Supplementary file 1 [file ijms-27-02928-s001.zip › ijms-4180712-supplementary.pdf]

Controlling *in vitro* mRNA polyadenylation by monitoring poly(A) polymerase consumption of ATP

Janja Skok[1¥], Pooja Tiwari[2¥], Tina Vodopivec Seravalli[1], Sergeja Lebar[1], Ana Ferjančič Budihna[1], Anže Martinčič Celjar[1], Polona Megušar[1], Matija Povh[1], Nina Mencin[1], Swapnil Bawage[2], Shree R. Singh[2], Artem Badasyan[3,*] and Rok Sekirnik[1,*]

[1] Sartorius BIA Separations d.o.o., A Sartorius Company, Mirce 21, 5270 Ajdovščina, Slovenia

[2] Arnav Biotech, 380 Michel St. NW, Atlanta, GA 30313, United States of America

[3] School of Science & Materials Research Lab, University of Nova Gorica, Vipavska 13, SI-5000 Nova Gorica, Slovenia, EU

[¥] Authors contributed equally

[*] To whom correspondence should be addressed: artem.badasyan@ung.si and rok.sekirnik@biaseparations.com

Supplementary Materials

GFP and Luc mRNA reporters are easy to detect but have limitations linked to short half-life, substrate dependence, and relatively low signal intensity. Variable heavy chain domain of a heavy chain antibody (VHH) mRNA has emerged as a promising alternative for evaluating delivery efficiency and formulation performance in mRNA delivery systems. It has been used in high-throughput in vivo screens to assess biodistribution and delivery efficiency across various models (1,2). VHH mRNA is also being repurposed for therapeutic applications, such as enhancing CAR T-cell targeting in tumor models (3) and enabling selective transfection of virus-specific T cells in vivo (4). This suggests VHH mRNA could potentially be used for gene therapy, vaccine development, and immunoengineering.

These studies rely solely on VHH-based staining and sequencing, which may lead to suboptimal screening results. To address this, mDeco was developed for efficient screening of different mRNA delivery methods. mDeco features a unique VHH that does not bind to mammalian, bacterial, or viral proteins and is expressed as anchored on the cell membrane with a glycosylphosphatidylinositol linker. It includes common tags, e.g. HA, V5, and c-myc to eliminate antibody affinity biases and streamline optimization protocols, important for in vivo studies. mDeco contains a TEV protease site and the expressed protein can be purified via His-tag. Fluorescent microscopy shows uniform cell surface staining of mDeco for various tags, with 90 % cells expressing mDeco by flow cytometry (Figure S1). MFI expectedly varied across tags due to fluorescence signature and intensity associated with the dye and other factors like cell type, staining protocol and compensation. This suggests that while VHH mRNA-based screening may yield suboptimal results, mDeco-based screening can enhance methods including immunohistology, western blot, dot-blot, immunoprecipitation, cell sorting, magnetic bead cell sorting/separation, ELISA, EIA, RNA-seq, and for this reason was used as a reporter mRNA in present study.

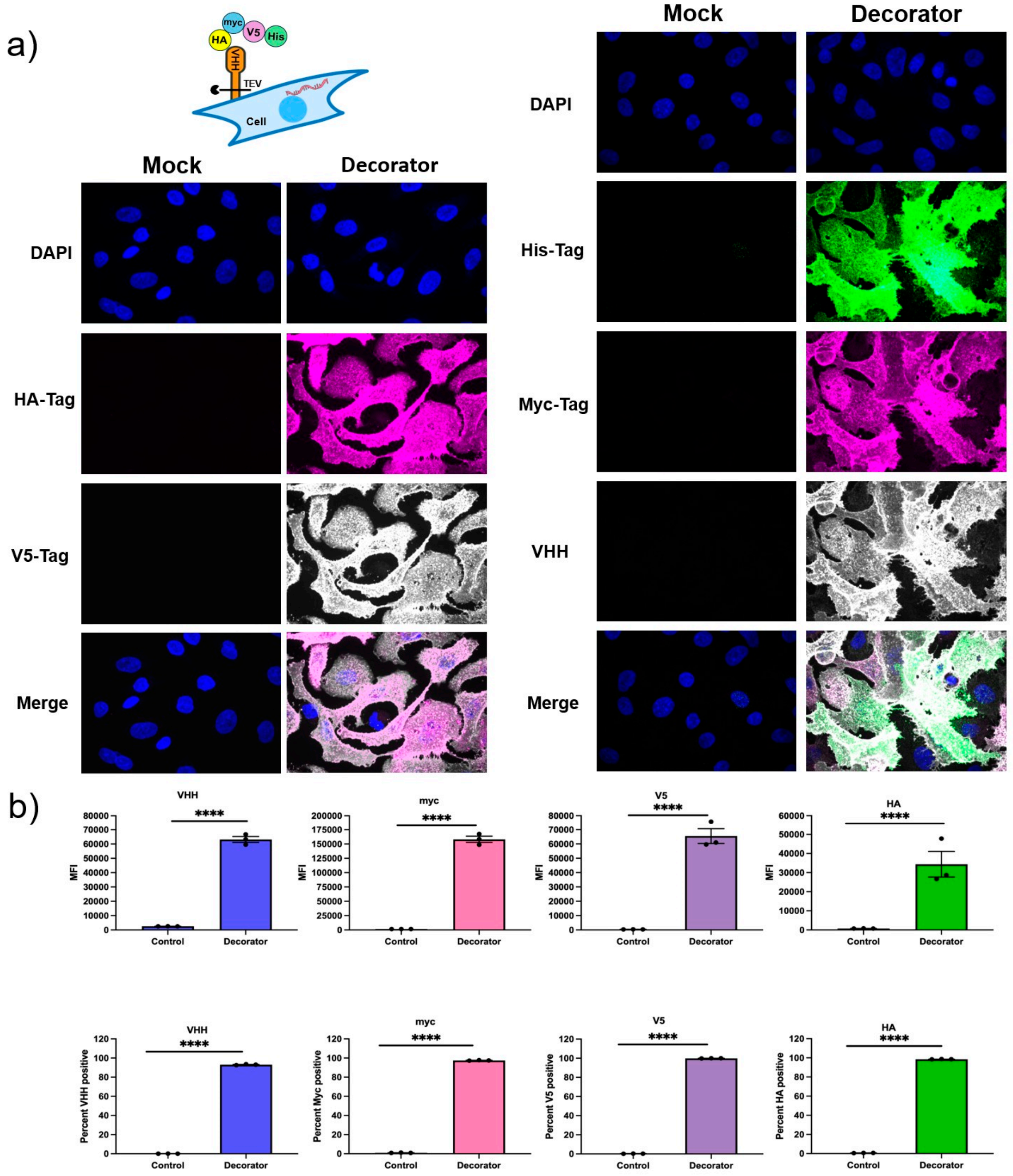

Figure S1: Schematic representation of mDeco mRNA expressed in cells and fluorescent microscopic images of A549 cells expressing mDeco (after 24h of transfection) that were stained for multiple tags and VHH (a) and flow cytometric analysis demonstrating mean fluorescence intensity (MFI) and percent cells expressing mDeco mRNA (b). Mock transfected cells do not show any staining by microscopy and flow cytometry. Data shown as the mean ± SEM (n = 3 biologically independent samples) with unpaired t-test analysis (B) with P values (*P < 0.05; **P < 0.01; ***P < 0.001; ****P < 0.0001).

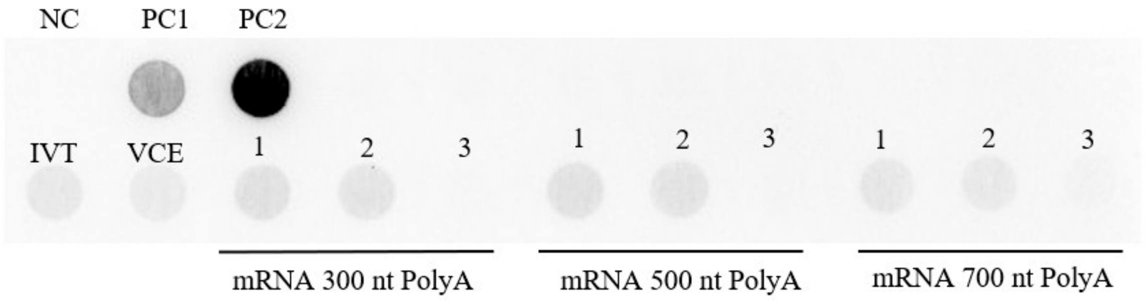

Figure S2: J2 dot-blot analysis of mDeco mRNA samples at different purification stages. mRNA was analyzed for presence of residual dsRNA at various stages throughout the production and purification process. No difference in J2 dot-blot signal intensity was observed between IVT, capping reaction (VCE), and polyadenylation reaction products (1). Samples were purified with Oligo dT (2) and SDVB column (3). Oligo dT purified mRNA samples exhibited comparable J2 dot-blot signal intensity between different poly(A) lengths. After SDVB purification, no J2 dot-blot signal was detected for any of the three poly(A) tail lengths. NC: negative control. PC: positive control Magi dsRNA standard (RNA Greentech, USA) at two mass loadings (PC1: 2 ng, PC2: 10 ng).

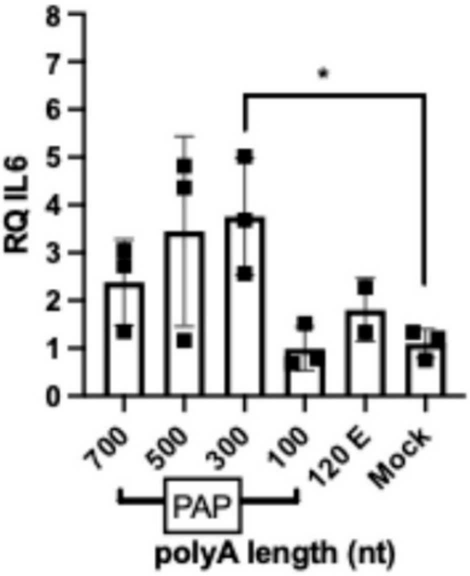

Figure S3: IL6 gene expression in A549 cell lines after 24hr of transfection with mDeco mRNA with PAP mediated 100 nt, 300 nt, 500 nt, 700 nt poly(A) tail and template encoded 120 nt poly(A) tail. The IL6 gene expression was normalized with GAPDH housekeeping gene, RQ for treatment groups were compared to the mock transfected group. Data shown as the mean ± SEM (n = 3 biologically independent samples) with unpaired t-test analysis with non-significant P values for all treatment groups compared mock group.

References

1. Hatit MZC, Lokugamage MP, Dobrowolski CN, Paunovska K, Ni H, Zhao K, et al. Species-dependent in vivo mRNA delivery and cellular responses to nanoparticles. Nat Nanotechnol. 2022 Mar;17(3):310–8.
2. Zenhausern R, Jang B, Schrader Echeverri E, Gentry K, Calkins R, Curran EH, et al. Lipid nanoparticle screening in nonhuman primates with minimal loss of life. Nat Biotechnol [Internet]. 2025 June 26 [cited 2025 Nov 26]; Available from: https://www.nature.com/articles/s41587-025-02711-y
3. Gamboa L, Zamat AH, Thiveaud CA, Lee HJ, Kulaksizoglu E, Zha Z, et al. Sensitizing solid tumors to CAR-mediated cytotoxicity by lipid nanoparticle delivery of synthetic antigens. Nat Cancer. 2025 May 16;6(6):1073–87.
4. Su FY, Zhao QH, Dahotre SN, Gamboa L, Bawage SS, Silva Trenkle AD, et al. In vivo mRNA delivery to virus-specific T cells by light-induced ligand exchange of MHC class I antigen-presenting nanoparticles. Sci Adv. 2022 Feb 25;8(8):eabm7950.
